# Infertility Challenge in a Cryptozoospermic Patient With Y Chromosome Microdeletion

**DOI:** 10.7759/cureus.59370

**Published:** 2024-04-30

**Authors:** Prerana Dagwar, Namrata Choudhary, Jarul Shrivastava, Krushnali S Kadu, Princee Tyagi, Akash More

**Affiliations:** 1 Clinical Embryology, Datta Meghe Institute of Higher Education and Research, Wardha, IND

**Keywords:** y chromosome microdeletion, testosterone, icsi, clomiphene citrate, cryptozoospermia, infertility

## Abstract

A couple is usually diagnosed with infertility if they have regular, unprotected sexual activity for a year or longer and are unable to conceive. Male infertility can be categorised into three types: obstructive infertility, non-obstructive infertility, and coital infertility. A major contributing factor for infertility in men is Y chromosome microdeletion, which is a non-obstructive infertility that involves problems related to sperm production. Deletions in the azoospermia factor region known as azoospermia factor a (AZFa), azoospermia factor b (AZFb), and azoospermia factor c (AZFc) loci independently or together which are situated on the Y chromosome cause a disturbance and alteration that are linked to either a reduction in sperm count, known as oligozoospermia, or the absence of sperm cells in the semen sample, referred to as azoospermia. Observations indicate that individuals with AZFc microdeletion may display irregularities in endocrine hormones. Men experiencing hormonal abnormalities affecting sperm production may receive treatment with clomiphene citrate. In cases of azoospermia and numerous cryptozoospermic patients, intracytoplasmic sperm injection is frequently considered the primary therapeutic approach.

## Introduction

Infertility is typically diagnosed when a couple engages in consistent, unprotected sexual activity for a year or more without successfully achieving conception [1]. The male partner causes infertility in 50% of the cases [2]. Infertility in males can be categorised into three main groups: non-obstructive infertility, involving issues with sperm production; obstructive infertility, relating to difficulties in sperm transit via the reproductive system; and coital infertility, which includes conditions that prevent erections and ejaculations [3]. Genetic defects may be interpreted for 15% of male infertility which is a non-obstructive infertility [2]. Klinefelter syndrome is the known and familiar hereditary reason for male infertility, which occurs due to the 47,XXY karyotype. Additional genetic causes include single-gene disorders, balanced translocations and other autosomal rearrangements, anomalies of the sex chromosomes in terms of number and structure, and single-gene mutations [2,4]. Male infertility is also significantly influenced by Y chromosome microdeletions which is a non-obstructive infertility. The rate of Y chromosome microdeletions in certain males having conditions such as non-obstructive azoospermia as well as severe oligozoospermia can reach up to 15% as well as 20% [2].

Disturbance and changes were caused because of Y chromosome microdeletions at the azoospermia factor a (AZFa), azoospermia factor b (AZFb), and azoospermia factor c (AZFc) regions of the azoospermia factor (AZF) between Yq11.21 and Yq11.23. AZFa, AZFb, and AZFc frequently have deleted areas in AZF in Y chromosome microdeletions. Deletions involving the entire AZFc gene are the most common, and their severity can lead to either severe oligospermia or azoospermia, and deletion involving the AZFa and AZFb genes results in severe azoospermia, where sperm retrieval is not possible [4,5]. Individuals with AZFc microdeletions have been found to exhibit endocrine hormone abnormalities [6]. Y chromosome microdeletions causing male infertility represent an example of a genetic condition detectable through polymerase chain reaction (PCR) screening. PCR results can be obtained rapidly, and the technique is relatively straightforward to utilize [7]. Cryptozoospermia is categorised as a type of virtual azoospermia according to the rules set forth by the World Health Organization [7]. In this case, sperm are found in the centrifuge sediment but are not visible when looking at the ejaculated semen under a microscope [8]. For many patients with cryptozoospermia and azoospermia, intracytoplasmic sperm injection (ICSI) is the main course of treatment [9].

This study focuses on the successful application of assisted reproductive technology to retrieve sperm from a patient with a genetic condition causing Y chromosome microdeletion and cryptozoospermia.

## Case presentation

To address infertility problems, a 32-year-old man along with his 27-year-old wife was recommended at a reproductive centre as the couple was unable to conceive following four years of marriage.

**Figure 1:**
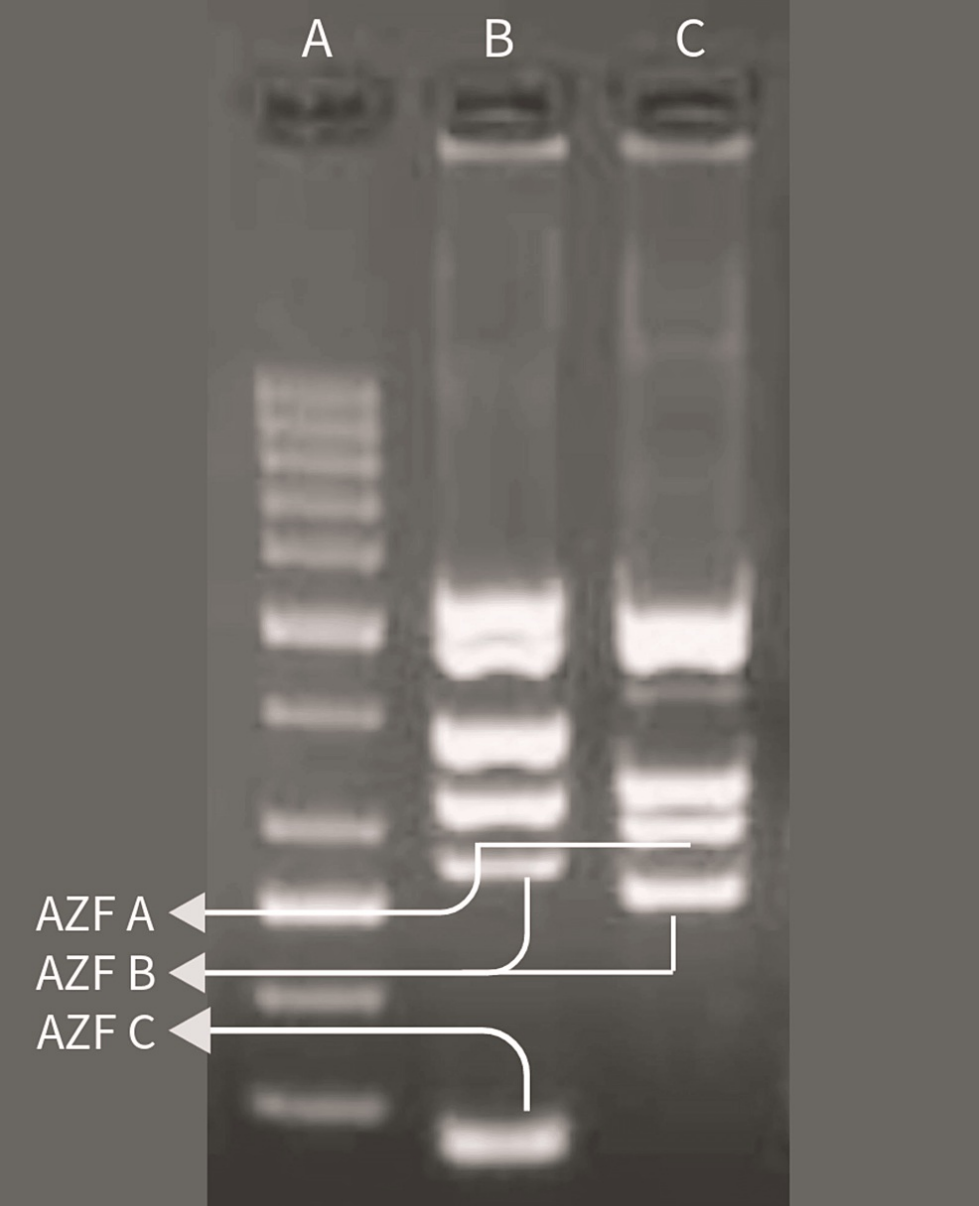
Agarose gel electrophoresis of PCR product PCR: polymerase chain reaction; AZFa: azoospermia factor a; AZFb: azoospermia factor b; AZFc: azoospermia factor c

The blood sample of the patient was collected at Wardha Test Tube Baby Centre, Acharya Vinoba Bhave Rural Hospital (AVBRH), Wardha, India, and sent to a laboratory for PCR to identify any missing genetic material on the Y chromosome. Also, the patient underwent hormonal testing to determine the levels of testosterone, luteinising hormone, and follicle-stimulating hormone in the patient's body. The follicle-stimulating hormone was 1.5 IU/L, the luteinising hormone was 0.9 IU/L, and the testosterone level was 1.8 ng/dL. The normal reference levels in male adults are as follows: luteinising hormone 1-8 IU/L, follicle-stimulating hormone 2-10 IU/L, and testosterone 2-8 ng/dL [10]. For the PCR, several sequence-tagged sites unique to the Y chromosome have been identified, and the primer sequences were released. PCR was carried out in 50 ul volumes, and for every primer at 1\iM, the primers amplified a DNA fragment. For three minutes, the PCR was carried out at 94°C. In that time, 35 cycles were carried out, and 60 seconds at 94°C, 60 seconds at 58°C, and 60 seconds at 72°C were completed [7]. Then, the absence or presence of Y chromosome microdeletion was determined by gel electrophoresis. The PCR interpreted disruption in the AZFc region of the patient's Y chromosome. In Figure 1, A indicates the ladder, and B shows normal control indicating the presence of AZFa, AZFb, and AZFc regions. In contrast, C shows the AZFa and AZFb region presences, and the AZFc region is found to be missing on the Y chromosome of the patient.

Subsequently, a semen analysis confirmed cryptozoospermia in the semen sample of the patient which is characterised by a low concentration of sperm. Despite processing the sample for ICSI, the oocyte failed to get fertilised.

In this case, primary infertility was caused because of non-obstructive cryptozoospermia, in which the underlying cause of infertility was the disruption specifically at the AZFc region of the patient's Y chromosome.

Treatment

The patient was prescribed to take clomiphene citrate tablets for 10 days to enhance testosterone level and sperm production in the patient's body. A sterile wide-mouth semen container was used to collect the patient's semen at the hospital. After the semen sample was liquefied, its volume was measured at 2 ml. The semen analysis was then conducted. The 2 ml semen sample was transferred to a fresh sterile round bottom tube, and 4 ml of sperm wash media containing protein supplements and antibiotics was added using a sterile pipette. The mixture was thoroughly combined and centrifuged at 1000 rotations per minute for 10 minutes. The supernatant was discarded, and 0.5 ml of sperm wash media with protein supplements and antibiotics was layered over the pellet. The processed sample was then prepared for ICSI. Subsequently, the sample was introduced into a polyvinylpyrrolidone (PVP) solution. Four oocytes were obtained from the female's ovaries through a minor surgical procedure and placed in a 4-(2-hydroxyethyl)-1-piperazineethanesulfonic acid (HEPES)-based medium. ICSI was then performed on the retrieved oocytes. Three oocytes were successfully fertilised. We conducted a fresh embryo transfer with blastocyst-stage embryos on the patient's spouse, and the procedure was successful. On day 14 of embryo transfer, the beta chorionic gonadotropin value measured 213 m IU/ml which indicates a positive pregnancy outcome.

## Discussion

In approximately 85% of cases, there is an identifiable cause for infertility. In 15% of infertile couples, there is "unexplained infertility" [11]. In 50% of cases of infertility, male infertility is majorly observed [2]. About 10% of men seem to have non-obstructive azoospermia, and 5% of men seem to have severe oligospermia; Y chromosome microdeletions are found within the AZF regions of the Y chromosome [12]. The Y chromosome holds the distinction as it is the smallest chromosome among the human chromosomes [13]. A centromere separates the two arms of the Y chromosome into one short arm (Yp) and one long arm (Yq) consisting of two pseudoautosomal regions (PARs). The Y chromosome is acrocentric [14]. The AZF region which is located at the sub-chromosomal level on the long arm of the Y chromosome and the AZF region on the Y chromosome which can be categorised into three segments from the chromosome's proximal to the distal end, identified as AZFa, AZFb, and AZFc, may undergo complete or partial deletion in Y chromosome microdeletion [15]. Y chromosome carries genetic information which is important for the spermatogenesis process and its regulation. There is a direct correlation between absent or partially impaired spermatogenesis and deletion in the AZF region of the Y chromosome [7]. Spermatogenesis is a process in which spermatogonial cells differentiate into spermatocytes through mitotic division and produce haploid spermatids from primary spermatocytes through meiotic division and ultimately give rise to spermatozoa, which occur within the testes [16].

Until recently, the Y chromosome was generally considered non-essential for life, with most of its regions assumed to be functionally inert [13]. Studies indicate that both reduced sperm count recognised as oligozoospermia and complete absence of spermatozoa in the semen, well known as azoospermia, are the result of deletions of AZF loci on the long arm of the Y chromosome. The genes essential for spermatogenesis and testicular development are housed within the AZF region [2,15]. Complete deletion in the AZFa region on the long arm of the Y chromosome leads to azoospermia as well as causes Sertoli cell-only syndrome. In this case, spermatozoa cannot be even retrieved through surgical procedures for ICSI. Similarly, the complete deletion of AZFb results in azoospermia as it leads to a spermatogenic arrest. In the case of complete AZFc deletions, individuals can have azoospermia to cryptozoospermia and oligozoospermia [14,17]. About 4% of men have partial AZFc deletions. These deletions are common in the male population [4,5]. Microdeletions in the AZFc region have been observed in normal men and are also another potential reason for reduced testosterone levels [18].

Clomiphene citrate operates as a selective estrogen receptor modulator which increases the secretion of follicle-stimulating hormone and luteinising hormone by altering the pituitary and hypothalamic estrogen receptors, and it lessens the negative effects of estradiol. The heightened levels of luteinising hormone contribute to the elevation of serum testosterone by acting on Leydig cells in the testis [19,20].

Cryptozoospermia is the term for spermatozoa concentrations in a man's ejaculate that are less than one million per millilitre [10]. Cryptozoospermia is often considered a special type of extreme oligozoospermia [8]. When there are extremely few or no spermatozoa in the ejaculate, fertilisation and pregnancy can still be achieved by the use of ICSI, which involves directly introducing a spermatozoon into an oocyte [13]. Despite the relatively high sperm recovery rate observed in individuals with AZFc chromosomal microdeletion, the outcomes of ICSI utilising retrieved sperm are still poor [17].

## Conclusions

Y chromosome microdeletions in males tend to have infertility as it causes non-obstructive azoospermia, severe oligozoospermia, and cryptozoospermia in certain males. In such cases, ICSI is the only ultimate choice for the fertilization of the oocyte, even though the chances of successful pregnancy through ICSI are not very assured in males with Y chromosome microdeletion genetic disorder. The male was prescribed medication to increase testosterone level as well as follicle-stimulating hormone and luteinising hormone level to increase sperm production as the male was found to have cryptozoospermia as well as low hormone level. After proper medication and treatment were provided to the patient, ICSI was carried out and pregnancy was achieved successfully.
